# Editorial: DNA Methylation Dynamics and Human Diseases

**DOI:** 10.3389/fcell.2022.956286

**Published:** 2022-06-23

**Authors:** Chunjie Jiang, Shengli Li

**Affiliations:** ^1^ Division of Diabetes, Endocrinology, and Metabolism, Department of Medicine, Baylor College of Medicine, Houston, TX, United States; ^2^ Precision Research Center for Refractory Diseases, Institute for Clinical Research, Shanghai General Hospital, Shanghai Jiao Tong University School of Medicine, Shanghai, China

**Keywords:** DNA methylation, next-generation sequencing, human diseases, 5-methylcytosine (5mC), cancer

DNA methylation is a biological process that adds methyl groups to DNA molecules. It regulates chromatin architecture and transcription, and plays essential roles in a wide range of biological processes (Li et al., 2013; Li et al., 2018; Roy et al., 2021). Accumulating evidence shows that the dysregulation of DNA methylation is involved in the development of many life-threatening diseases, including cancers, cardiovascular diseases (Pepin et al., 2019; Heery and Schaefer, 2021; Pepin et al., 2021; Dillinger et al., 2022; Rosselló-Tortella et al., 2022). This Research Topic aims to elucidate the correlation between DNA methylation and human diseases, with the hope to deepen our understanding of the underlying molecular mechanisms of DNA methylation in human diseases and pave the way to the development of new strategies or methods for disease prevention, diagnosis, and therapy. In total, there are 9 original research articles that have been published in this Research Topic.

In this Research Topic, we received articles in the fields of cancer biology, cardiac biology and covid research. Adding methyl groups to the C-5 position of cytosine, 5-methylcytosine, is the main type of DNA methylation (Choi et al., 2021), whose dysregulation has been reported to play roles in many types of cancer. To understand the 5-methylcytosine in cancer, one of the primary cause of death worldwide (Siegel et al., 2020), Liu et al. developed a 5-methylcytosine score system and evaluated tumor mutation burden, immune check-point inhibitor response, and the clinical prognosis of individual tumors using the score based on the 5-methylcytosine profile of 1,374 lung adenocarcinoma samples. Besides, by analyzing 5-methylcytosine and gene expression profiles from 860 clear cell renal cell carcinoma (ccRCC), Xu et al. found LINC00861 was the potentially intervening target of immunotherapy for prostate cancer patients and was significantly associated with the expression of PD-1 and CTLA4. Li et al. developed predictive diagnostic and prognostic models by using machine-learning and Cox regression approaches based on pancreatic adenocarcinoma (PAAD) datasets. To take the advantage of the third-generation sequencing technique, Zhang et al. collected two pairs of tumor tissues and adjacent normal tissues from hepatocellular carcinoma (HCC) surgical samples, and then conducted Nanopore sequencing. Zhang et al. identified four potential tumor suppressor genes (KCNIP4, CACNA1C, PACRG, and ST6GALNAC3) by the integrative analysis of 5-methycytosine and 6-methyladenine profiling. Their study provided a new solution for epigenetic regulation research and therapy of HCC. LncRNAs have been shown to have high tissue- and disease-specific expression patterns, which endows them the potential in therapy (Wapinski and Chang, 2011; Jiang et al., 2016; Jiang et al., 2019). Zhao et al. focused on lncRNAs differentially expressed in only one and multiple cancer types, and identified 29 lncRNAs as diagnostic biomarkers for ccRCC, the kidney renal papillary cell carcinoma (KIRP), and pan-cancer.

Dysregulated regulation of miRNAs has been shown to contribute to the pathogenesis of cardiovascular diseases (Shao et al., 2015; Dong et al., 2020; Qiu et al., 2020; Yang et al., 2020). To investigate the interaction between miR-29b and DNA methylation in cardiovascular diseases, Wu et al. collected heart tissue samples from 17 patients with congenital heart disease (CHD). The authors found an inversely correlation between miR-19a and DNA methyltransferases (DNMT) in the patients. Further exploration in hypomethylated zebrafish showed that miR-29b inhibitor relieved the deformity of hypomethylated zebrafish and restored the DNA methylation patterns in cardiomyocytes, resulting in increased proliferation and renormalization of gene expression, suggesting a mutual regulation between miR-29b and DNMTs in cardiomyocytes and supporting the miRNA-based therapy in cardiomyocytes.

Coronavirus disease 2019 (COVID-19), caused by severe acute respiratory syndrome coronavirus 2 (SARS-CoV-2), has become a global public health crisis. To explore the roles of 5-Hydroxymethylcytosine in COVID-19, Chen et al. developed a machine learning model based on genome-wide 5-Hydroxymethylcytosine profiles in plasma cell-free DNA (cfDNA) from 53 healthy volunteers, 66 patients with moderate COVID-19, 99 patients with severe COVID-19, and 38 patients with critical COVID-19. They found 5-Hydroxymethylcytosine detected in cfDNA could be used as early warning markers for the disease progression and myocardial injury of COVID-19.

In summary, by taking the advantage of the state-of-the-art high-throughput sequencing technologies, the authors showed the function role of DNA methylation in various types of cancer, as well as in cardiovascular disease and COVID-19. We would like to thank all authors for their paper published in this Research Topic. These studies made significant contributions in the field, extending our understanding of the roles of DNA methylation in human disease and will facilitate further advancement. Nevertheless, studies investigating the dynamic changes of DNA methylation of human diseases and the potential of DNMT in disease therapeutics are still lacking, which might be the directions of future efforts in this Research Topic. In addition, the rapid development of sequencing technologies, such as nanopore DNA sequencing, will accelerate our research and discovery in this field.
